# The Impact of Culture on Access to and Utilisation of Maternity Care Amongst Muslim Women in High‐Income Countries: A Qualitative Systematic Review

**DOI:** 10.1111/1471-0528.18290

**Published:** 2025-07-22

**Authors:** Aljawharah Al‐Mubarak, Brana Ahilan, Tisha Dasgupta, Sergio A. Silverio, Hiten D. Mistry, Lojain Al‐Harbi, Jawza Aldakhail, Stephanie Heys, Peter von Dadelszen, Laura A. Magee

**Affiliations:** ^1^ Department of Women & Children's Health School of Life Course & Population Sciences, King's College London London UK; ^2^ King Abduallah International Medical Research Centre Riyadh Saudi Arabia; ^3^ Department of Psychology Institute of Population Health, University of Liverpool Liverpool UK; ^4^ Department of Population Health Sciences University of Leicester Leicester UK; ^5^ National Health Emergency Operation Centre Ministry of Health Riyadh Saudi Arabia; ^6^ Wiliam Harvey Research Institute Queen Mary University of London London UK; ^7^ The North West Ambulance Service Manchester UK

**Keywords:** culture, ethnic minority, meta‐ethnography, Muslim, postpartum, pregnancy, qualitative research, systematic review, women

## Abstract

**Background:**

Global human migration has highlighted the need to provide culturally appropriate maternity care, delivered in accordance with the recipient's beliefs and practices.

**Objectives:**

This review aims to examine the impact of culture on access, utilisation, and care delivery of care for Muslim women during pregnancy and postpartum through the experiences of women, families, and maternity care‐providers.

**Search Strategy:**

Six electronic databases were searched for published qualitative and mixed‐methods studies, in English (01/January/2003‐12/October/2023).

**Selection Criteria:**

Studies undertaken in high‐income countries report the experiences of either Muslim women accessing and utilising maternity services, or healthcare professionals delivering those services.

**Data Collection and Analysis:**

Meta‐ethnography was used to develop new concepts from included studies.

**Main Results:**

Of 23 428 articles identified, 24 met the inclusion criteria. Four themes were identified: ‘Religious influences’, ‘Sociocultural interactions’, ‘Healthcare as a culture’, and ‘Disrupted communication’. Women's negative experiences highlighted cultural insensitivity, providers' unconscious bias, inflexible care models (and the conflict between expectations of services and those offered), and cultural stereotyping in addition to indifferent and uniform care. Healthcare professionals' experiences highlighted challenges with miscommunication and Muslim women's reliance on information (and sometimes, misinformation) from their communities.

**Conclusions:**

Our findings highlight the challenges involved in delivering culturally‐sensitive care to Muslim women; issues which extend beyond the confines of culture‐specific awareness of religion and ethnicity, to the universal concept of personalisation. This is reflected in the theory, ‘Recognise our differences, embrace our diversity, and care for me as an individual’.

**Registration:**

International Prospective Register of Systematic Reviews (PROSPERO): CRD42024499304

## 1 Introduction

Global migration has necessitated the provision of maternity care to populations of diverse cultures and with varied experiences [1, 2, 3]. Yet, studies continue to highlight that women from ethnic minority (vs. White) backgrounds experience poorer pregnancy outcomes, including higher maternal morbidity and mortality rates [2, 3, 4, 5, 6].

These heightened risks of maternal complications amongst ethnic minority women may be attributed, partly, to delays in accessing maternity care or its under‐utilisation [2, 3, 5]. Barriers include transportation difficulties and financial instability [1, 2, 3, 4, 5, 6, 7]. A recent qualitative systematic review extends this list of barriers for ethnic minority women from high‐income countries (HICs) by adding poor health literacy, prior negative experiences, and cultural insensitivity, which were reported as contributing to delaying initiation of antenatal care (ANC) or failing to complete recommended ANC visits [3].

Cultural insensitivity in healthcare is described in literature as “the inability to be appropriately responsive to the attitudes, feelings, or circumstances of groups of people that share a common and distinctive racial, national, religious, linguistic, or cultural heritage” [8]. This insensitivity stems from differences between the cultures of care‐users and care‐providers, at the level of individual healthcare professionals (HCPs) and broadly, the healthcare system [5]. Cultural insensitivity may have a negative impact on care‐seeking through mistrust and poor quality of care, especially where implementation of culturally appropriate maternity care is challenged by linguistic barriers and lack of resources, including staff training [9, 10, 11].

The present systematic review aims to synthesise the qualitative literature focused on Muslim women, and both the receipt and provision of culturally appropriate maternity care, defined as that which is delivered in accordance with the recipient's religious or sociocultural beliefs and practices [3, 4, 12, 13].

## 2 Methods and Study Design

### 2.1 Protocol and Registration

The review was prospectively registered with the International Prospective Register of Systematic Reviews [14]. This systematic review was conducted according to the Preferred Reporting Items for Systematic reviews and Meta‐Analyses (PRISMA) guidelines [15]. Whilst PRISMA was used to guide the reporting of our literature search and study selection process, the eMERGe reporting guidance was consulted to inform the reporting of our meta‐ethnographic synthesis, including the processes of translation, synthesis, and theory generation. This dual approach allowed us to ensure both methodological transparency and fidelity to the interpretive nature of meta‐ethnography [15, 16, 17].

### 2.2 Eligibility Criteria

Our search strategy was designed to identify the global literature on the impact of cultural factors on access to, utilisation, and delivery of maternity care. We identified all relevant qualitative studies (i.e., interviews, focus groups, or free text responses in surveys) evaluating the experiences of pregnant women (and their families) receiving maternity care, and those of HCPs providing that care. We defined ‘culture’ as a group's beliefs and practices that define them, related to issues that include (but are not limited to) language, religion, custom, social norms, and laws [1, 2, 3, 4, 5, 6, 7, 9]. Our outcome of interest was the experience of care receipt (by women and families) or provision (by HCPs), evaluated in a qualitative study design. To eliminate additional influences, such as economic and conflict issues which may not directly relate to care provision or receipt, studies were only eligible if conducted in HICs (according to the 2023–2024 World Bank index).

### 2.3 Searches

A preliminary search was conducted to identify all relevant search terms, using keywords and synonyms, based on ‘Population‐Exposure‐Outcomes’ (PEO) criteria as listed in Table S1.

Six electronic databases were searched, from January 2003 to 27 November 2023: Clinical Index for Nursing and Allied Health Literature (CINAHL), Medline (Ovid), Global Health (Ovid), Maternity, Infant Care database (MIDIRS, Ovid), Embase (Ovid), and Scopus. PubMed and Google Scholar were used to search citation and reference lists. Our search was limited to English publications. A detailed description of the search strategy is shown in Table S2.

### 2.4 Study Selection

Identified articles were uploaded to the Rayyan software [18]. Following the removal of duplicates, team members (AA‐M, BA, JA, LA‐H) independently screened the titles and abstracts of identified articles. Abstracts of studies which contained insufficient information were moved to full‐text review, undertaken independently by two reviewers (AA‐M, BA) for final article inclusion. The reference lists of included articles were reviewed for additional potential publications to consider for inclusion. Any conflicts were resolved by discussion. In the event of unresolved conflict, a third reviewer (LAM, PvD, SH) was consulted.

Given the broad range of literature identified and the multiple representations of culture, the included studies were grouped prior to data extraction, then categorised to either ‘stable’ or ‘migrant’ populations. Articles meeting inclusion criteria which studied stable populations, focused on ethnic/racial minority status, indigenous populations, and sociocultural vulnerabilities (such as homelessness or adolescent pregnancy). Articles focusing on migrant populations, focused on ethnicity, sociocultural vulnerabilities (such as asylum‐seekers and undocumented refugees), as well as religion and female genital mutilation (FGM). This study focused on Muslim women, and included publications focused on participants from the Middle East, where there are predominantly Muslim populations.

### 2.5 Quality Assessment

Two reviewers independently assessed the quality of included studies, using an adaptation of the Walsh and Downe tool for qualitative research appraisal [19]. This tool assesses research quality based on scope and purpose, design, sampling strategy, analysis, interpretation, reflexivity, ethical dimension, and relevance and transferability [19]. To best fit our study, we amended an adaptation of the tool by Yuill et al. [20], which reduced the number of items in the original Walsh and Downe tool and utilised a numeric scoring system instead of a categorical system. The resultant tool consisted of 35 items, each scored from 0 to 2 (with 0 representing poor, 1 representing moderate, and 2 representing high quality), and the total score ranging from 0 to 70, with 0–24 as low overall quality, 25–47 as moderate, and 48–70 as high. No studies were excluded based on study quality (Table S3).

### 2.6 Data Extraction and Synthesis

Data from included articles were extracted independently by two reviewers (AA‐M, BA), using a predesigned form in Microsoft Excel (Table S4).

Following data extraction, each paper was uploaded to NVivo, a computer‐assisted qualitative data analysis software used to assist researchers in managing, organising, visualising, and reporting their data [21]. Participants' accounts (quotations) from the results sections were highlighted in the uploaded paper to be coded for an analytic synthesis.

Reviewers (AA‐M, BA) undertook a meta‐ethnography, as a seven‐phase analytic‐synthesis technique for qualitative data to explore potential new understandings beyond the findings of the original studies, once papers are amassed and participants' data from different studies can be pooled [16]. This method was determined appropriate to conceptualise experiences and perceptions related to the provision and receipt of maternity care by Muslim Women and understand the determinants of their access and utilisation of care.

The meta‐ethnography analysis was conducted in accordance to the eMERGe reporting guidance as follows: First‐order constructs (i.e., quotations, the primary qualitative data) and second‐order constructs (i.e., primary authors' interpretations) were first coded, and then the codes were combined to create a hierarchical cluster of similarities (reciprocal data) and differences (refutational data) across included studies analysing similarities and differences first within studies relating to women's experiences then the experiences of HCP's [17]. These clusters were compiled to generate third‐order constructs or a ‘line of argument’ the reviewers' interpretations from a tertiary analysis of first‐ and second‐order constructs, combining findings from both categories. The results were presented as descriptive themes relating to the relevant experiences of patients and HCPs [16, 17].

## 3 Results

### 3.1 Literature Search

**FIGURE 1:**
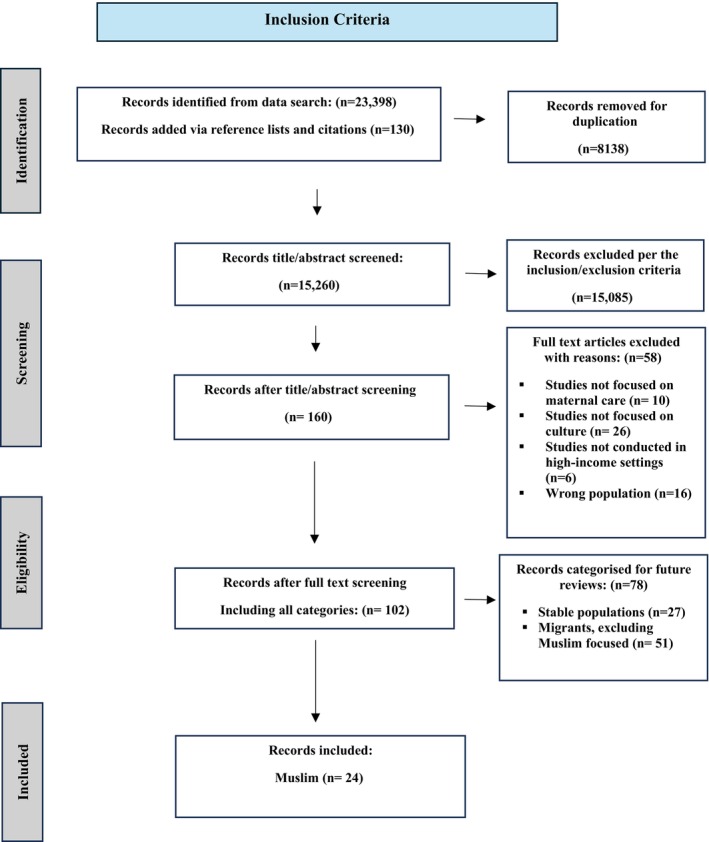
The Preferred Reporting Items for Systematic reviews and Meta‐Analyses (PRISMA) flow diagram of screening, selection process and inclusion study.

Of 23 528 articles identified, 102 relevant articles met eligibility criteria, of which 24 focused on Muslim women [16, 22, 23, 24, 25, 26, 27, 28, 29, 30, 31, 32, 33, 34, 35, 36, 37, 38, 39, 40, 41, 42, 43, 44, 45], and were included in this review (Figure 1).

### 3.2 Study Quality Assessment

Most studies (*n* = 23) were of high quality with an average score of 61/70 [22, 23, 24, 25, 26, 27, 28, 29, 30, 31, 32, 33, 34, 35, 36, 37, 38, 39, 40, 41, 42, 43, 44], with one study of moderate quality with a score of 41/70 [45] (Table S4).

### 3.3 Study Characteristics

Most studies were conducted in Europe (*n* = 14) [22, 23, 26, 28, 29, 30, 31, 32, 33, 34, 35, 36, 37, 41, 42, 43, 44], with the remaining from North America (*n* = 9) [24, 27, 33, 36, 38, 39, 40, 44, 45], or across two settings (Norway and Australia) [25]. Studies were carried out from 2002 to 2018.

Most (*n* = 19) undertook interviews for data collection [23, 24, 25, 26, 27, 28, 29, 30, 31, 32, 35, 36, 37, 38, 39, 40, 43], whilst three studies used focus group discussions [22, 33, 45] and two studies used both [34, 37].

Most studies focused on women's experiences of receiving maternity care (*n* = 22) [22, 23, 24, 26, 27, 28, 29, 30, 32, 33, 34, 35, 36, 37, 38, 39, 40, 41, 42, 43, 44, 45], whilst two studies involved partners [22, 44] and another two studies explored experiences of HCPs in delivering care [25, 31]. There were 373 participants, of which 293 were women; 45 were maternity HCPs, most of whom were midwives; and 35 were male partners and family members. Most participants were either self‐identified as, or were suggested to be, Muslim women [22, 23, 25, 27, 29, 32, 33, 34, 35, 36, 37, 41, 42, 43, 44, 45]; only eight studies explicitly stated participants' religion [24, 26, 28, 30, 31, 38, 39, 40]. For further details of individual studies, see Table S5.

Whilst our findings inform the experiences of Muslim women with maternity care in HICs, which may be applicable to many Muslim women around the globe, it is to note that these findings were synthesised based on a selected sample of Muslim women.

### 3.4 Synthesis and Findings

**FIGURE 2:**
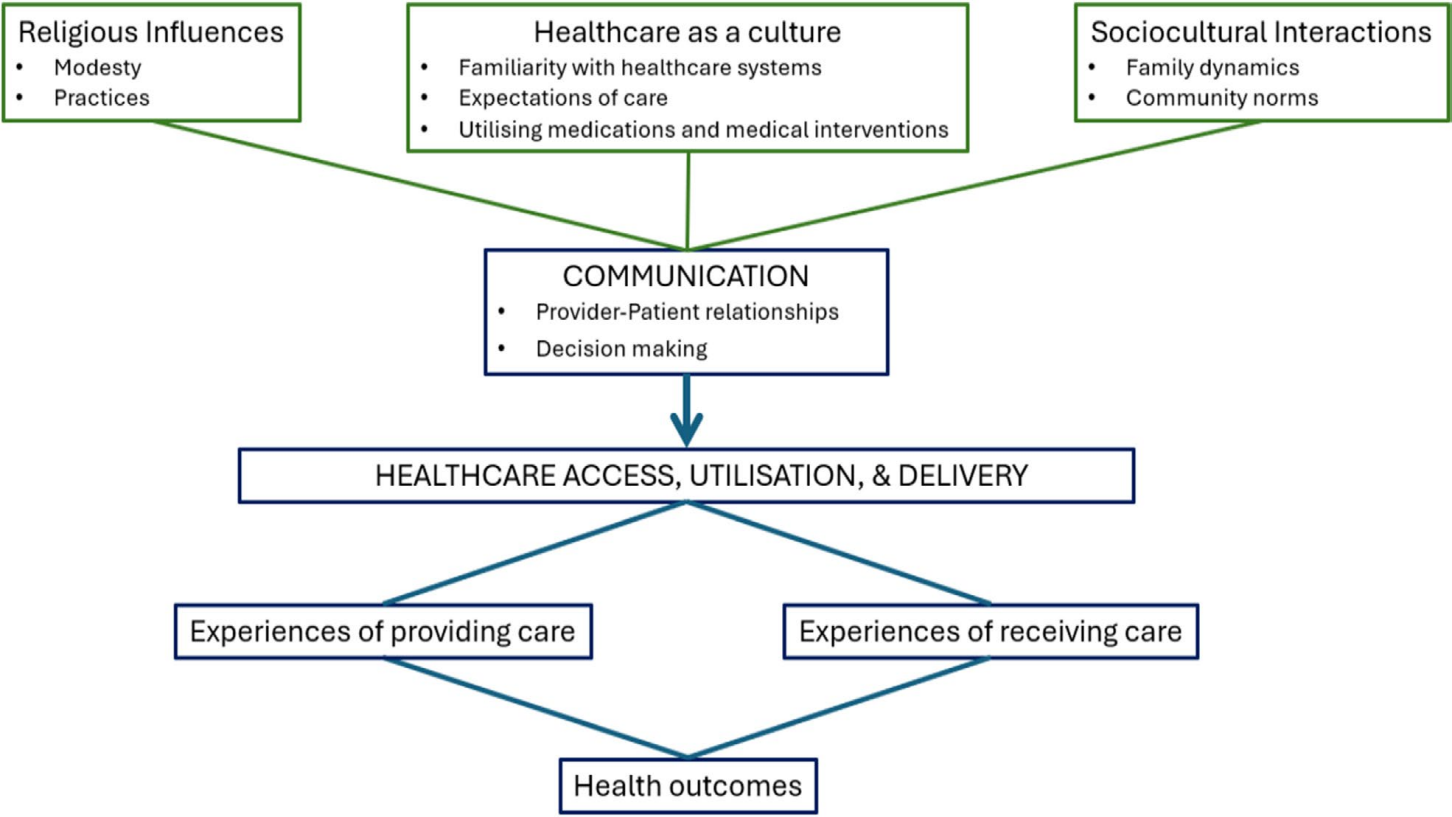
The diagram presents the identified themes and their sub‐themes showcasing cultural related barriers to Muslim women and family's access and utilisation of maternity care as well as cultural related challenges to maternity healthcare professionals delivering care to Muslim and Middle Eastern women.

Meta‐ethnography produced four themes (‘Religious influences’, ‘Sociocultural interactions’, ‘Healthcare as a culture’, and ‘Disrupted communication’), and nine sub‐themes (Figure 2).

#### 3.4.1 Theme 1: Religious Influences

This theme reflected the experiences of women (*n* = 9) [24, 27, 28, 30, 32, 38, 39, 40, 44] and HCPs (*n* = 3) [22, 25, 31], and had two sub‐themes: ‘Modesty’ and ‘Religious Practices’ (Table 1). Negative experiences of women were heavily influenced by their cultural practices and religious beliefs.

**TABLE 1:** ‘Religious influences’ theme, sub‐themes, and supportive quotations.

Sub‐themes	Authors' interpretations of original findings	Examples of quotations from study participants
Modesty	**Women**	
	Gender preferences to health care‐providers (HCPs') and modesty [22]	“I choose to go with a female [doctor]. That's more comfortable for me, actually. That's what feeling more comfortable, you can do your best.” (Transcript 35) [22]
	**Healthcare professionals**	
	HCPs' understanding and awareness of religious practices [29]	“[Previously] I was certain that Muslim women would judge the situation [a critical or not critical] appropriately [when making the decision about their gender preference of the HCP]. [However], one scenario where a Muslim woman did not want a male doctor. She was bleeding internally; she had an accident but would not let a male doctor touch her. I think perhaps most people would think that would be horrendously stupid” (HCP‐5—White British) [29]
Religious Practices	**Women**	
	Culture affecting care interactions [26]	“[Then I thought to myself]: ‘If I want to listen to the Quran, I will do that. Why do you react this way [i.e., contemptuously]? You do not have to find it right. You do not have to find it correct. But please keep your opinion to yourself. Do not show what you think of it!’ It really was not the moment. Outside [in another context], he [gynaecologist] can talk to me about it, but not while I'm giving birth! I'm not saying that he should respect it, that he should think it is a good idea. No. Maybe according to him it is totally useless, I can agree with that. But why react so negatively? […] You [as a caregiver] must be able to reassure someone who is giving birth. You should not give her a negative feeling about it [her delivery].” [26]
	**Healthcare professionals**	
	HCPs' understanding and awareness of religious practices [29]	“I do not think you can ever be so direct because then you are hitting a line, where she [Muslim woman] thinks you are judging her and you are being rude. I think it is always about asking, have you been fasting?” (HCP‐7—Asian‐bilingual) [29]

Abbreviation: HCPs, healthcare professionals.

##### 3.4.1.1 Modesty

Women's interactions with HCPs were regulated by their Islamic teachings; this affected care utilisation, as most women preferred female HCPs. Whilst this was especially true when physical examination was required [24, 30, 38, 39, 40], women were also embarrassed discussing pregnancy‐related matters with male HCPs [24, 30, 32, 42], through male interpreters [27, 42, 43], or whilst attending mixed‐gender group prenatal classes [24, 44]. Participants preferred a more private setting [24, 44] to discuss intimate details of their reproductive health [24, 30, 40, 42, 44]. Of note, some Muslim women revealed they simply preferred female HCPs for personal reasons [39, 43]. Failure to respond to Muslim women's desire to have a female HCP left women feeling upset [24, 30, 38, 44]. Conversely, attempts to accommodate women's religious needs had a positive impact on their experiences, and when their beliefs were considered, women were more likely to accept care delivered by male professionals, if necessary [32, 38, 39, 40].

HCPs found that when Muslim women requested treatment by a female provider, this often interfered with care delivery [25, 31]. When a female HCP was not available, pregnant women faced delays in receiving care [25, 31].

##### 3.4.1.2 Religious Practices

Muslim maternity care‐users considered their religious practices as important, especially during labour [28, 30, 38]. These practices include fasting, praying the Shahadah (a profession of faith prayer) and reciting the Quran. Unsupportive attitudes towards these expressions of religion made women reluctant to disclose relevant information (e.g., fasting during pregnancy), potentially having a negative impact on care [28, 30, 38, 39]. In contrast, a few women appreciated HCPs showing respect for their beliefs and integrating those beliefs into their care, and this improved their care experience [28, 42].

Some HCPs reported finding it difficult to ask if their patients were fasting, due to fear of offending, whilst others expressed the view that even when asked, most Muslim women did not divulge fasting, for fear of being judged [31].

#### 3.4.2 Theme 2: Sociocultural Interactions

This theme reflected the experiences of women and families (*n* = 11) [27, 28, 29, 33, 34, 36, 37, 39, 43, 44, 45], and HCPs (*n* = 3) [22, 25, 31], with two sub‐themes: ‘Family dynamics’ and ‘Community norms’ (Table 2).

**TABLE 2:** ‘Social interactions’ theme, sub‐themes, and supportive quotations.

Sub‐themes	Authors' interpretations of original findings	Examples of quotations from study participants
Family dynamics	**Women**	
	Trust in the experiences of and advice from community members [32]	“My mother has been through it before, so I ask her questions. I do not bother going to the doctors, I put it off. I do not see the midwives often. Any concerns or worries that I might have I might call on someone experienced who has had 4 children and ask. There are however, a lot of myths and you have to separate them from facts.” (Interview 2) [32]
	**Healthcare professionals**	
	Healthcare providers perceptions about Muslim women [29]	“They [Muslim women] learn from their mothers and aunties and sisters, things like breastfeeding, they [Muslim women] just automatically will breastfeed their babies and do it well” (HCP‐1‐ White British) [29]
Community norms	**Women**	
	Religious, personal, and traditional/community influences, views and experiences [32]	“In our community we tell each other things. My cousins are more educated, some have more kids than I do, so they know what they are talking about; they have more real‐life experiences.” (Participant 2, FG2) [32]
	**Healthcare professionals**	
	Role of religious and cultural influences [23]	“It is important to find links in their community because they are only with us for such a short time” (A7) [23]

##### 3.4.2.1 Family Dynamics

Muslim women felt family members should be the primary source of information during pregnancy and postpartum [29, 33, 34, 37]. Reliance on familial advice was particularly strong when women did not receive adequate information from their HCPs [39]. Some women felt that only new mothers, first‐time pregnant women, may need to attend antenatal visits or participate in prenatal classes because knowledge acquired from family members provides them with sufficient guidance [29, 33, 34, 37]. However, other women acknowledged the need to consult maternity HCPs in addition to seeking family advice [45].

Healthcare professionals described family influences on care access and utilisation [22, 25, 31]. In a cross‐country study in Sweden, midwives found the participation of families to be positive, whilst Australian midwives found family influences to hinder care delivery [25]. In both studies, HCPs noted that women's reliance on family advice often interfered with medical recommendations [22, 25, 31]. Moreover, input from male family members was perceived as a barrier to delivering personalised care as women's needs may not be properly articulated [22, 25, 31].

##### 3.4.2.2 Community Norms

As with family members, women found community members to be a source of support, especially after immigration, when no family was present during pregnancy [28, 36, 38]. Some women stated that many women in their communities seek maternity care services only when in labour or when there is an emergency [34, 43, 44].

Healthcare professionals noted Muslim women's dependence on support from other women in the community had contributed to delays in accessing care [25, 31]. As such, some suggested that providing midwives of a similar culture to pregnant women might encourage them to use maternity services [31].

#### 3.4.3 Theme 3: Healthcare as a Culture

This theme reflected the experiences of women and families (*n* = 15) [22, 23, 24, 28, 29, 30, 32, 33, 34, 35, 38, 39, 40, 41, 44], and HCPs (*n* = 3) [22, 25, 31], with three sub‐themes: ‘Familiarity with healthcare systems’, ‘Expectations of care’ (relevant only to women and families), and ‘Utilising medications and medical interventions’ (Table 3).

**TABLE 3:** ‘Healthcare as a culture’ theme, sub‐themes, and supportive quotations.

Sub‐themes	Authors' interpretations of original findings	Examples of quotations from study participants
Familiarity with healthcare systems	**Women**	
	Knowledge of the UK healthcare system [21]	“You must be aware of your rights; you must stand [up] for your rights. If you do not, you will be easily ignored. No one works as a volunteer to help you. So, I missed a lot of maternity benefits.” (Participant number 3) [21]
	**Healthcare professionals**	
	Building knowledge with Somali women about pregnancy care [23]	“It seems as though they don't much like interventions and yes, including some medicines for example. Rather they want things to be natural and it's the same if they go over time, and we usually get the labour going at 42 + 0, but they often really want to wait and let it happen naturally so to speak. And they don't count on the fact that we here in Sweden, we're so bound up with dates and times [for doing things]. So, on the surface there's this broad sense of being relaxed but in fact if there's time and you get to know people there are many social issues underneath there that women are carrying and managing” (S5) [23]
Expectations of care	**Women**	
	Conflicting Views on Treatment [24]	“When I knew I was pregnant I went to the GP to make sure. I thought they would do some tests for it, as happens in my country. The GP only asked me when my last period was, and he decided I was pregnant even though I always had irregular periods.” (Kawther) [24]
Utilising medications and medical interventions	**Women**	
	Health system challenges [20]	“For Swedish speakers we have parent education [in groups], yes. Not for non‐Swedish speakers, then we give education individually, in the consultation room. And it becomes shorter, and not the same amount.” (Midwife 1 FGD 7) [20]
	**Healthcare professionals**	
	Health care‐providers' understanding and awareness of religious practices [29]	“I do not think you can ever be so direct because then you are hitting a line, where she [Muslim woman] thinks you are judging her and you are being rude. I think it is always about asking, have you been fasting?” (HCP‐7—Asian‐bilingual) [29]
	Challenges in the midwife‐parent encounter: dealing with stereotypes [20]	I think it is frustrating when we don't understand, why don't they comply with our recommendations? And this is a pretty complicated issue, this issue of women who shouldn't get pregnant again but they do. But this applies to other smaller things as well like coming late and this issue of [not taking] iron tablets… (Midwife 1 FGD 7)

##### 3.4.3.1 Familiarity With Healthcare Systems

Several women felt lost and confused trying to navigate healthcare systems different from those in their country of origin; this led to missed opportunities to access appropriate services [23, 24, 29, 35, 38, 42, 44]. Participants expressed their experiences with maternity care services could have been better if they had received proper guidance regarding available resources [24, 26, 44]. However, migrant care‐users noted that the longer they resided in their host country, the more familiar they became with the healthcare system, and this improved their access and utilisation of maternity care [22, 44].

Healthcare professionals recognised the need to familiarise women with available services, to encourage care access and utilisation. They further noted that providing such education was a positive experience [22, 25, 31]. Midwives expressed women's lack of familiarity with routine care protocols led to disruption in care [22, 25].

##### 3.4.3.2 Expectations of Care

Due to a lack of familiarity, multiparous participants expected their care to be similar to previous care, which resulted in dissatisfaction with the care received [24, 28, 32, 33, 35, 40]. Women in Arab countries reported maternity care services to be more flexible and accommodating compared with care in the UK, where unscheduled visits were not felt to be a common practice [26]. Women reported feeling abandoned when their expectations of care were not consistent with the care delivered [26]. Across several studies, Somali women were apprehensive about accessing Western maternity care, where Caesarean birth is more common; they regarded medicalised birth as a poor outcome for mothers and their fertility [29, 33, 34, 36, 41, 44].

##### 3.4.3.3 Utilising Medications and Medical Interventions

Both women and HCPs felt that conflicting views on treatment were due, at least in part, to cultural differences between healthcare user and provider [22, 25, 31]. Under those circumstances, when the offer of such intervention was declined, this often made HCPs worried for women's health and safety [22, 25, 31]. Australian midwives noted that even when informed of available interventions, women often chose healthcare practices familiar to them, such as natural remedies [25].

For prenatal testing for chromosomal abnormalities, some women explained their rejection of testing for fear this might lead to advice to terminate the pregnancy, which they emphasised was against their beliefs [24, 30, 37]; others simply did not want to feel anxious about the potential results [29, 33, 34, 41, 43, 44].

For women who had undergone FGM, all aspects of maternity care were affected. Women expressed to their HCPs the need for de‐infibulation (re‐opening the vagina) to avoid pain and tearing during childbirth; however, women felt the procedure was rarely provided due to the lack of familiarity with FGM and its management, leaving women traumatised following childbirth [33]. Care‐providers acknowledged their inability to deliver advice or adjust care when they were not knowledgeable about certain cultural practices [22, 25, 31]. Caesareans were reported to have been offered for women with FGM, even when it was not needed, due to their lack of training in de‐infibulation procedures [41]. Again, women had positive experiences when providers were culturally aware and attentive to their needs [28, 44].

#### 3.4.4 Theme 4: Disrupted Communication

This theme explores how cultural differences between healthcare users and HCPs may lead to disruptions in communication which have negative consequences on care access, utilisation, and delivery (*n* = 18) [22, 23, 26, 27, 28, 29, 30, 32, 33, 34, 35, 36, 37, 38, 39, 40, 42, 45]. There were two sub‐themes: ‘Provider‐patient relationship’ and ‘Decision‐making’ (reported only by women) (Table 4).

**TABLE 4:** ‘Disrupted communication’ theme, sub‐themes, and supportive quotations.

Sub‐themes	Authors' interpretations of original findings	Examples of quotations from study participants
Provider‐patient relationship	**Women**	
	Cultural awareness and preconceived ideas [32]	“When you have that kind of attention (being ignored) you feel you are a burden and thus tend to avoid seeking help from the people you feel you are a burden on. You keep everything to yourself and want to get out of the place quickly.” (Interview 04) [32]
	**Healthcare professionals**	
	Challenges in building rapport: language and communication issues [23]	“The disadvantage with an interpreter is that it's not the same as talking to the woman yourself. Often there can be misunderstandings and sometimes it's hard to know. I have had lots of experiences where the woman says ‘Yes, yes’, but next time I want to be sure she had followed the recommendations I've given or if she has any questions. And then I realise she hasn't understood anything, and I don't know if she just wanted to be polite when she said ‘Yes, yes’. It's hard” [23]
Decision‐making	**Women**	
	Childbirth education needs [43]	“They always ask me to choose amongst the different pain medications like epidural anaesthesia and the other pain medications, without full explanations of their side effects.” [43]

##### 3.4.4.1 Provider‐Patient Relationship

A perceived lack of cultural awareness amongst care‐providers made women fearful of being judged and hesitant to openly communicate with care teams and specifically about services offered [22, 33, 34, 36, 43, 45]. Some women chose not to engage with services such as antenatal classes because they were not adequately informed about them [24, 34]. Women with FGM felt humiliated by providers' attitudes during physical examination [41, 43]. As a result, there were negative pregnancy experiences [28, 29, 30, 32, 42, 45]. However, women who were cared for by providers familiar with their culture reported feeling less isolated [35, 41, 42].

Language proficiency and health literacy affected patient‐provider relationships [22, 28, 34, 35, 42]. Women felt HCPs should and could be respectful and caring, regardless of language barriers [37]. Providers agreed and highlighted there are other forms of effective communication and highlighted the need for cultural awareness training [22, 25, 31]. A lack of interpretation services often delayed care utilisation [25, 32, 34, 41], and many women felt that using interpreters was ineffective and interfered with patient‐provider confidentiality [23, 26, 43]. Similarly, HCPs found language barriers challenging, particularly with regard to building a rapport with women [22, 25, 31].

##### 3.4.4.2 Decision‐Making

Participants preferred to be informed about their care and to actively participate in decision‐making [27, 32, 43, 45], to maintain their autonomy [28, 32, 44]. Lack of information, miscommunication, misunderstanding, and language barriers all impaired care‐users' ability to share in decision‐making [22, 27, 40, 41, 43, 44], and made women more sceptical about the medical advice received [34, 36, 42, 44]. They were more inclined to trust and follow medical direction from a provider acquainted with their culture [23, 44]. Of note, however, was that some women felt comfortable leaving decision‐making to their care‐providers [40]. Those women were used to a patriarchal healthcare system and trusted their providers to do what is best for their care [40].

## 4 Discussion

### 4.1 Main Findings

This qualitative systematic review included 24 studies of Muslim women's views on the receipt of maternity care, as well as HCPs' views on their provision of said care. The four themes derived focused on religious influences, sociocultural interactions, the healthcare system as a culture, and disrupted communication.

Women's experiences with maternity care services were positive when communication was open and care was appropriate to the individual and their cultural needs, and negative when these were not the case, which often led to refusal or delayed use of services offered. Women's negative experiences highlighted cultural insensitivity and stereotyping, providers' unconscious bias, inflexible care models, and difficulty with language barriers. However, their largely negative experiences were not caused exclusively by culturally inappropriate care, but also by care which was indifferent, uniform, and not personalised. Care‐users' past experiences of maternity care in their home countries highlighted how the healthcare system culture in host countries impacts women's interaction with maternity care services, a theme that was not highlighted in the included literature.

Studies of providers' perspectives on delivering care to Muslim women were limited to only three studies [22, 25, 31]. There were particular challenges with miscommunication rooted in cultural differences, and a reliance of Muslim women on information from their own communities. Importantly, providers' experiences varied between women, despite their cultural similarities. Providers acknowledged the need for cultural training to improve women's experiences and suggested a co‐production strategy between women and providers [22, 25, 31].

### 4.2 Interpretation

Our findings highlight the challenges involved in delivering culturally‐sensitive care to Muslim women, but also that issues extend beyond the confines of culture‐specific awareness of religion and ethnicity, to the universal concept of personalisation. This is reflected in the theory: *‘Recognise our differences, embrace our diversity, and care for me as an individual’.*

Our findings are consistent with global literature on inequalities, including Muslim women. Immigrant women from ethnic minorities have more positive maternity care experiences when they have a better understanding of the language and the setting of the host country where they migrate [2, 3, 6, 12]. The importance of communication has been emphasised repeatedly, as has the negative impact of discriminatory behaviour on care‐seeking [46]. Similar to our findings, other research has found women tend to rely on familial advice as a result of negative experiences with maternity care‐providers [5, 9, 47].

Other studies presented similar findings to our review regarding the challenges that care‐providers face when caring for Muslim women during pregnancy and childbirth, including cultural differences and communication barriers [9, 47, 48, 49]. Preconceived ideas about immigrant care‐users were apparent amongst the included studies, and impacted the way care was delivered [48], as well as the physical and legal implications of certain cultural practices (i.e., FGM) [50]. It is apparent from our findings, consistent with the literature, that ‘culture‐clash’ between women and providers remains a barrier to care.

Our findings are consistent with the broader healthcare literature on the interaction between personal characteristics and sociocultural factors [51]. Factors such as culture are indirectly associated with global disparities in utilisation and provision of maternity care, as well as maternal mortality and morbidity outcomes [51]. Studies have highlighted delays in initiating care due to women lacking information about available services [2, 3, 12, 47, 52, 53, 54]. On the other hand, there is extensive literature on interventions to reduce maternal health inequalities by educating women, designing services to involve the community, implementing cultural competency training for maternity care‐providers, and mobilisation of HCPs with similar backgrounds to care‐users [54].

Negative attitudes of HCPs have been highlighted as a deterrent for women to access and utilise maternity care services [3, 4, 7, 12]. Rigid Western healthcare systems have been identified as being largely ill‐suited to the individual needs of non‐Western and migrant women [53]. In Western high‐income settings specifically, a lesser regard for non‐Eurocentric communities compounds the impact of these disparities in maternity and other care [55]. Reviews have emphasised that services that involve the community have increased women's access to maternity care [5, 9, 47]. Mobilised HCPs with similar linguistic and cultural backgrounds, to collaborate with a multi‐disciplinary team of midwives, physicians, and psychologists, have proved effective in improving patient satisfaction, engagement with maternity care services, and knowledge about pregnancy, birth, and parenthood for > 88% of users [56].

Whilst employing care‐providers of similar culture to Muslim women may assist in minimising culture‐clash between care‐users and care‐providers, it is difficult to envision this as a comprehensive solution, given staffing challenges where Muslim women do not form the predominant culture and diverse needs must be met [9, 48]. Relatedly, our findings also highlighted that personalised women‐centred care is the core for achieving a culturally appropriate care delivery within maternity services. The review showed that women's representation of their cultural and religious values varied, impacting their access and reception of care. That said, there appears to be clear justification for having care‐providers skilled at de‐infibulation.

Lastly, Muslim women's experiences are variable and influenced by other factors such as education, economic status, and personal ideations, which can be observed in wider literature as some experiences resonated with vulnerable and marginalised women of other communities while others experienced challenges of a different nature.

### 4.3 Strengths and Limitations

Strengths of our study include a comprehensive literature search which included all stakeholder groups. Although several reviews have discussed the experiences of ethnic minority women with maternity services, we believe that ours is the first to focus specifically on the impact of cultural factors on Muslim women and to explore the topic from the perspectives of women and maternity care‐providers. Also, and uniquely, we have used a meta‐ethnography approach which extends the findings of the included studies themselves by allowing for new interpretations.

Limitations of our review are inherent in the primary literature on which the review is based. There are a limited number of published studies exploring care‐providers' experiences of providing care to Muslim women, and those studies do not detail providers' demographics, ethnicity, and gender, making it challenging to ascertain generalisability to the workforce in similar settings.

## 5 Conclusions

This systematic review has identified considerable scope for improvement in culturally appropriate maternity care of Muslim women, from care‐user and care‐provider perspectives. These informed that respectful care is at first a moral standard as well as a professional requirement which should be upheld within the healthcare system. Strategies to maintain such a standard must consider a personalised way of care to inclusively address culture‐specific issues, employing co‐production services to ensure a better experience and care delivery.

Adopting a lens of ‘cultural safety’ will allow services to acknowledge differences and empower patients' rights in defining what “safe” care is during an encounter [57]. Although challenging to implement, especially with the directive approach health services use in implementing changes in practice, sharing the process of policy making with participants can reflect better care for women of minority groups.

Co‐production must therefore be at the heart of future solutions, particularly as some issues raised by Muslim women are common to the maternity care experiences of women more generally [5, 9, 12, 22, 25, 31, 47].

## Author Contributions

A.A.‐M., L.A.M., S.H. and P.v.D. planned the review including conceptualising the study selection criteria; A.A.‐M. searched the literature and screened eligible articles. B.A., L.A.‐H. and J.A. also screened the articles. A.A.‐M. and B.A. undertook the data synthesis and manuscript draft. A.A.‐M., L.A.M., S.H., P.v.D., S.A.S., T.D. and H.D.M. contributed to manuscript writing.

## Ethics Statement

The authors have nothing to report.

## Conflicts of Interest

The authors declare no conflicts of interest.

## Supporting information


Table S1

Table S2

Table S3

Table S4

Table S5

## Data Availability

The data that supports the findings of this study are available in the Supporting Information of this article.
