# Supplementary material for: The Impact of Culture on Access to and Utilisation of Maternity Care Amongst Muslim Women in High‐Income Countries: A Qualitative Systematic Review
Source: BJOG. 2025 Jul 22;132(13):1996–2008. doi: 10.1111/1471-0528.18290 (PMC12592763; doi:10.1111/1471-0528.18290)
Supplement: Supplementary file 1 — Table S1 [file BJO-132-1996-s001.docx]

**Table 1. PEO criteria**

| Population:  Pregnant/postpartum women | (‘expectant mothers’, ‘expectant women’, or ‘childbearing’). |
| --- | --- |
| Or maternity HCP | Or (’midwives’ or ‘midwifery’ or ‘nurse’ or ‘obstetric’ or ‘obstetrician’). |
| Exposure:  Maternity care | (‘maternal services’, ‘obstetrics’, ‘perinatal care’, ‘postnatal care’, ‘postpartum care’). |
| Outcome:  Experiences; | (‘perceptions’ or ‘attitudes’) |
| relating to cultural factors | related to cultural factors (of ‘access’, ‘barrier’, ‘minorities’, ‘cultural awareness’, ‘cultural appropriateness’, ‘cultural competency’, ‘cultural sensitivity’, or ‘cultural responsiveness’). |
