# Supplementary material for: The Impact of Culture on Access to and Utilisation of Maternity Care Amongst Muslim Women in High‐Income Countries: A Qualitative Systematic Review
Source: BJOG. 2025 Jul 22;132(13):1996–2008. doi: 10.1111/1471-0528.18290 (PMC12592763; doi:10.1111/1471-0528.18290)
Supplement: Supplementary file 2 — Table S2 [file BJO-132-1996-s002.docx]

**Table S2**: Search strategy by electronic database searches and search dates covered

| **CINAHL**  (1981- 27 NOV 2023) | Ovid search strategy – includes:  **Medline** (1997 – 27 NOV 2023)  **Global Health** (1973 – 27 NOV 2023, wk 39) **Maternity and Infant Care Database** (MIDIRS, 1973 - 27 NOV 27 NOV 2023, wk 39) | Ovid  (**Embase**, 1974- 27 NOV 2023, wk 39) | **Scopus**  (2004 – 27 NOV 2023) |
| --- | --- | --- | --- |
| 1. Pregnancy or pregnant or prenatal or antenatal or perinatal or maternal or postnatal or postpartum or birth or mother or maternal or postnatal 2. maternity care' or 'antenatal care' or 'perinatal care' or 'obstetric care 3. 1 and 2 4. midwifery or midwives or midwife or nurse or nursing or maternity 5. obstetrician or obstetric or ob-gyn 6. doula or doula care or doulas or continuous labour support 7. 4 and 5 and 6 8. access or accessibility or seeking or barriers or obstacles or challenges 9. ethnic minorities or racial minorities or religious minorities or ethnic groups or religious groups 10. cultural competence or cultural awareness or cultural competency or cultural sensitivity 11. 8 and 9 and 10 12. experiences or perceptions or attitudes or views or feelings or perspective 13. qualitative research or qualitative study or qualitative methods or interview or focus group or discussion ethnographic or mix method 14. 3 and 8 and 11 and 12 and 13 | 1. Pregnan* or Matern* or Labo* or Deliver* Birth* or childbirth* or postnatal. 2. Emerg* or Prehospital* or EMS or Care* or Midwi* or Obstetri* or Paramed* 3. Cultur* or Experi* Percep* or cultural appropri* or cultural compet* or cultural sensit* or cultural respon* or cultural aware* or qualitative or focus group* or interview* 4. 1 and 2 and 3 5. Pregnan*.ti 6. Pregnan*.ab 7. Emerg*.ti 8. Emerg*.ab 9. Cultur*.ti 10. Cultur*.ab 11. 5 OR 6 12. 7 OR 8 13. 9 OR 10 14. 4 AND 11 15. 4 AND 12 16. 4 AND 13 17. Remove duplicates.   Limits: English language, human and humans.  Filtered by year 2003-2023. | 1. Pregnan* or Matern* or Labo* or Deliver* Birth* or childbirth* or postnatal. 2. Emerg* or Prehospital* or EMS or Care* or Midwi* or Obstetri* or Paramed* 3. Cultur* or Experi* Percep* or cultural appropri* or cultural compet* or cultural sensit* or cultural respon* or cultural aware* or qualitative or focus group* or interview* 4. 1 and 2 and 3 5. Pregnan*.ti 6. Pregnan*.ab 7. Emerg*.ti 8. Emerg*.ab 9. Cultur*.ti 10. Cultur*.ab 11. 5 OR 6 12. 7 OR 8 13. 9 OR 10 14. 4 AND 11 15. 4 AND 12 16. 4 AND 13 17. Remove duplicates.   Limits: English language, human and humans.  Filtered by year 2003-2023 | 1. Pregnan* or Matern* or Labo* or Deliver* Birth* or childbirth* or postnatal. 2. Emerg* or Prehospital* or EMS or Care* or Midwi* or Obstetri* or Paramed* 3. Cultur* or Experi* Percep* or cultural appropri* or cultural compet* or cultural sensit* or cultural respon* or cultural aware* or qualitative or focus group* or interview* 4. 1 and 2 and 3 5. TITLE-ABS-KEY (Pregnan*) 6. TITLE-ABS-KEY (Birth*) 7. TITLE-ABS-KEY (Emerg*) 8. TITLE-ABS-KEY (cultur*) 9. 5 OR 6 10. 7 OR 8 11. 4 AND 9 12. 4 AND 10   Limits: year 2003-2023, subject area of medicine, nursing, health professions, psychology, and social sciences, English language, human and humans,  Additional limitations: any studies before 2002 will be excluded. Search will be limited to articles. |
