# Supplementary material for: The Impact of Culture on Access to and Utilisation of Maternity Care Amongst Muslim Women in High‐Income Countries: A Qualitative Systematic Review
Source: BJOG. 2025 Jul 22;132(13):1996–2008. doi: 10.1111/1471-0528.18290 (PMC12592763; doi:10.1111/1471-0528.18290)
Supplement: Supplementary file 3 — Table S3 [file BJO-132-1996-s004.docx]

**Table S3**: Walsh and Downe quality appraisal tool (condensed)

| Study number | Study Author/year | Scope & purpose  (of 6) | Design  (of 32) | Interpretation  (of 8) | Relevance & transferability  (of 14) | Reflexivity (of 6) | Ethical dimension  (of 4) | Total  (of 70) |
| --- | --- | --- | --- | --- | --- | --- | --- | --- |
| 1 | M. Ahrne, E. Schytt, E. Andersson, et al. (2019). | 5 | 24 | 7 | 13 | 6 | 4 | 59 |
| 2 | Alshawish, Eman, et al. (2013) | 5 | 30 | 8 | 9 | 6 | 4 | 62 |
| 3 | Alzghoul, Manal M., et al. (2021) | 4 | 30 | 7 | 11 | 6 | 4 | 62 |
| 4 | Andersson, E., Nazanin, S., Estefania, O., & Small, R. (2021). | 6 | 30 | 6 | 13 | 6 | 4 | 65 |
| 5 | Bawadi, Hala, Zaid Al-Hamdan, and Muayyad M. Ahmad. (2020) | 5 | 27 | 7 | 8 | 2 | 4 | 53 |
| 6 | Cameron, Emma Stirling, et al. (2022) | 6 | 27 | 8 | 13 | 5 | 4 | 63 |
| 7 | Degrie, Liesbet, et al. (2020) | 6 | 32 | 8 | 14 | 6 | 4 | 70 |
| 8 | Glavin, Kari, and Berit Sæteren. (2016). | 6 | 23 | 7 | 13 | 4 | 4 | 57 |
| 9 | Hassan, Shaima Mohamed, Conan Leavey, and Jane S. Rooney. (2019) | 5 | 30 | 8 | 13 | 5 | 4 | 65 |
| 10 | Hassan, Shaima Mohamed, et al (2020) | 5 | 31 | 8 | 13 | 5 | 4 | 66 |
| 11 | Henry, Julia, Christian Beruf, and Thomas Fischer. (2020) | 6 | 28 | 8 | 10 | 4 | 4 | 60 |
| 12 | Herrel, Nathaly, et al. (2004) | 3 | 22 | 5 | 5 | 2 | 4 | 41 |
| 13 | Hill, Nancy, Emmy Hunt, and Kristiina Hyrkäs. (2012) | 6 | 29 | 8 | 14 | 6 | 4 | 67 |
| 14 | Konje, Joan K., and Justin C. Konje (2021).‏ | 5 | 30 | 8 | 14 | 5 | 4 | 66 |
| 15 | Lukin, Tanja-Tatiana, et al. (2023) | 6 | 29 | 8 | 12 | 6 | 2 | 63 |
| 16 | Missal, Bernita, Connie Clark, and Mariya Kovaleva. (2016) | 5 | 28 | 8 | 13 | 4 | 4 | 62 |
| 17 | Ny, Pernilla, et al. (2007) | 5 | 26 | 8 | 10 | 4 | 4 | 57 |
| 18 | Qureshi, Rubab, and Dula F. Pacquiao. (2013) | 6 | 28 | 4 | 8 | 4 | 4 | 54 |
| 19 | Reitmanova, Sylvia, and Diana L. Gustafson. (2008) | 5 | 28 | 8 | 11 | 4 | 4 | 60 |
| 20 | Simpson, Jennifer L., and Kimberly Carter. (2008) | 5 | 28 | 8 | 12 | 6 | 4 | 63 |
| 21 | Straus, Lianne, Andy McEwen, and Faduma Mohamed Hussein. (2009) | 5 | 28 | 8 | 12 | 6 | 4 | 63 |
| 22 | Utne, Renate et al. (2020) | 3 | 30 | 8 | 12 | 6 | 4 | 63 |
| 23 | Wallmo, Susanne, Karin Allgurin, and Carina Berterö. (2020) | 5 | 28 | 8 | 13 | 4 | 4 | 62 |
| 24 | Wojnar, Danuta M. (2015) | 5 | 27 | 8 | 12 | 4 | 4 | 60 |
