# Supplementary material for: The Impact of Culture on Access to and Utilisation of Maternity Care Amongst Muslim Women in High‐Income Countries: A Qualitative Systematic Review
Source: BJOG. 2025 Jul 22;132(13):1996–2008. doi: 10.1111/1471-0528.18290 (PMC12592763; doi:10.1111/1471-0528.18290)
Supplement: Supplementary file 4 — Table S4 [file BJO-132-1996-s003.docx]

# **Table S4:** Data extraction sheet


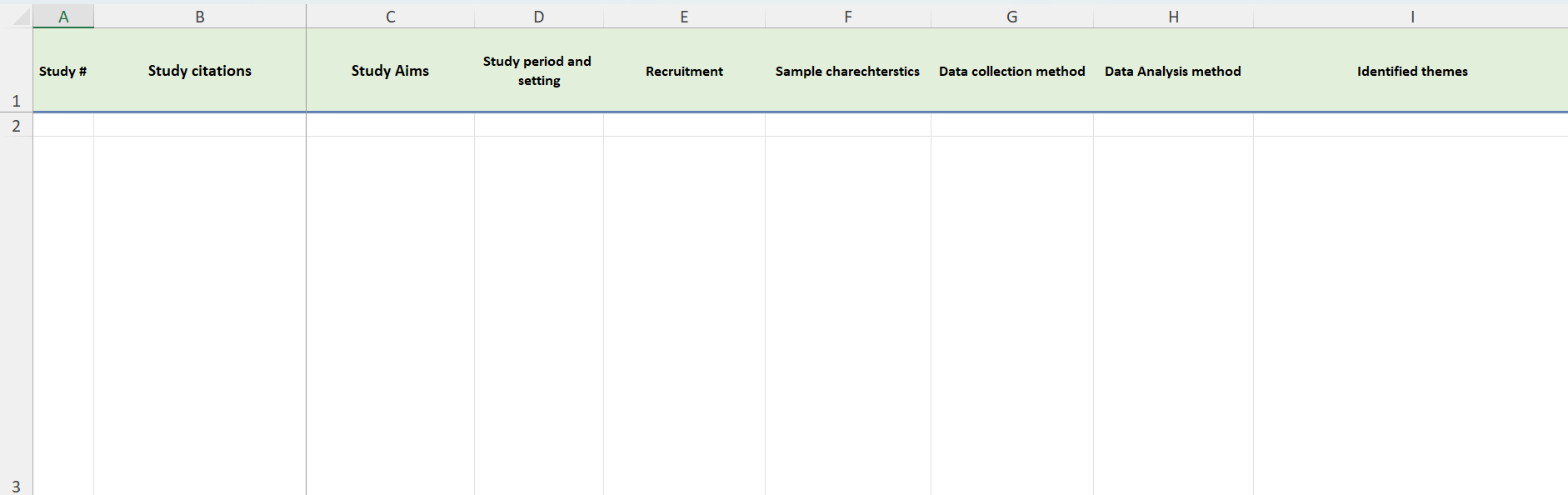


| Study No. | Study citation | Study aims | Study period and setting | Recruitment | Sample characteristics | Data collection method | Data analysis methods | Identified themes |
| --- | --- | --- | --- | --- | --- | --- | --- | --- |
|  |  |  |  |  |  |  |  |  |
