# Supplementary material for: The Impact of Culture on Access to and Utilisation of Maternity Care Amongst Muslim Women in High‐Income Countries: A Qualitative Systematic Review
Source: BJOG. 2025 Jul 22;132(13):1996–2008. doi: 10.1111/1471-0528.18290 (PMC12592763; doi:10.1111/1471-0528.18290)
Supplement: Supplementary file 5 — Table S5 [file BJO-132-1996-s005.docx]

**Table S5:** Summary of the characteristics of included studies.

| Author, year | Study aims | Study period and setting | Recruitment | Study sample | Data collection method | Data analysis method | Identified themes |
| --- | --- | --- | --- | --- | --- | --- | --- |
| Ahrne (2019) | To explore Somali-born mothers’ and fathers’ experiences of antenatal care in Sweden, antenatal care midwives’ experiences of caring for Somali-born parents, and their respective ideas about group antenatal care (gANC) for Somali-born parents | December 2016 and May 2017 (Sweden) | Purposeful sampling was used.  Midwives recruited at three ANC clinics.  Parents recruited via the Somali diaspora, public preschools, and Child Health Centres | Midwives (n=7) all female.  Parents:  (n=16) Somali female with one mother born in Sweden with Somali backgrounds,  (n=13) Somali male | Focused group discussions.  Midwives FGD (n=3),  Mothers FGD (n=2)  Fathers FGD, (n=3) | Thematic analysis: Attride-Stirling’s tool “Thematic networks” | A-Challenges in the midwife-parent encounter: (i) Tailoring care to individual needs (ii) Dealing with stereotypes (iii) Addressing variations in health literacy (iv) Overcoming communication barriers (v)Partner involvement. B-Health system challenges: (i)Accessibility (ii) Clear but flexible routine (iii) Limited resources (iv) Structures for parent education (v) Group antenatal care – one way forward? |
| Alshawish, (2013) | To investigate the access to and use of health services, particularly maternal and child health care, in the UK by Palestinian women. | Not stated (United Kingdom) | Women were recruited by identifying eligible Palestinian women in Arabic schools (via their children) and leaving invitation letters in the local mosque snowball sampling from the identified sample | Palestinian women (n=22) living in Manchester | In-depth semi-structured interviews | Framework analysis. | (i) cultural variations (ii) knowledge of the NHS and the UK health-care system (iii)health-care services and their utilisation (GP's and maternal and Childcare services) (iv) communication |
| Alzghoul, (2021) | To explore the perinatal health care experiences of Muslim women in NWO | Not stated (Canada) | Purposeful and snowball sampling from community centres | Muslim women (n=19) | Semi-structured interviews | Thematic analysis | (i) Women’s choices and preferences (ii) Attitudes toward prenatal classes and education. (iii)Husbands’ involvement and support in the birthing process. (iv) Challenges in receiving optimal care |
| Andersson, (2021) | To explore and compare how midwives in Melbourne and Stockholm experienced providing maternity care for Somali women | Not stated (Australia, Sweden) | Purposeful sampling. Swedish sample: community antenatal clinics. Australian sample: antenatal clinic of a large tertiary maternity hospital | Swedish midwives (n=8), Australian midwives (n=10) | Semi-structured interviews | Thematic analysis | (i) Midwives’ attitudes towards Somali women and their family and cultural  context (ii) Challenges in building rapport (iii) Overcoming challenges to improve car |
| Bawadi, (2020) | To examine the challenges faced migrant Arabic Muslim women in accessing maternity services, and to suggest ways to positive childbirth experience for them | Not stated (United Kingdom) | Purposeful sampling from religious institutions and community centres | Arab Muslim women (n=8) | Longitudinal Semi-structured interviews: (i)antenatal period  (ii) 1-2 weeks postpartum (iii)1-3 months postpartum | Interpretative phenomenological analysis | The Vulnerable Woman: (i) language exclusion (ii) discrimination and prejudice (iii) bureaucracy and “stiff upper lip” (iv)conflicting ideology (v) the conventional as strange. |
| Cameron, et al. (2022) | To understand the lived experiences of access to postnatal services and supports among refugee women who have resettled in Nova Scotia, Canada. | Not stated (Canada) | Purposeful sampling from community centres, health clinics, and private sponsorship organizations | Syrian refugee women (n=11) | Semi-structured interviews | Constructive grounded theory | (i) The importance of social support; (ii)impact of structural barriers on access to and quality of care (iii)presence of provider paternalism (iv) valued and missing services |
| Degrie, et al. (2020) | To fill this gap by presenting the first part (patients’ perspective) of the results of a large-scale qualitative research study about the intercultural care experiences of Muslim women from Turkish and Moroccan descent, as well as of their caregivers in maternity wards in Flanders, Belgium | May 2016-December 2017 (Belgium) | Purposeful sampling via key organisations, key persons, and informal get togethers in addition to snowball sampling and advertisements via a key organisation website and 6 relevant social media groups | Muslim women (n=24), of Turkish descent (n=11), of Moroccan descent (n=13) | Semi-structured interviews | Grounded theory | A- Ereignis and Erlebnis interplay (i) Protocolized care, (ii) uncaring care, (iii) Embraced care, (iv) Ambiguous care. B- Culture as a meaning system (i) Culture affecting the women’s expectations (ii) Culture affecting the care interactions (iii) Culture affecting the interpretation and coping of the experiences. |
| Glavin, Kari, et al. (2016) | To explore Somali new mothers’ experiences with the Norwegian health care system and the mothers’ experienced needs during their hospital stay and the postpartum period. | January 2014- August 2015 (Norway) | Purposeful sampling via two well-child clinics (Wwc's) | Somali Immigrant women (n=10) | Semi-structured interviews | Griesheim and Lundman’s framework analysis | (i) Inadequate integration into Norwegian society (ii) Need for and fear of caesarean delivery (iii) Family support around the postpartum period (iv) Support from health services |
| Hassan, Shaima et al. (2019) | To generate insight into Muslim women’s maternity experiences, consider their access to and experiences of the NHS maternity services, and the religious factors that influence their health seeking decisions | Not stated (United Kingdom) | Purposeful sampling via local mosque and local Muslim women community groups | Muslim women (n=7) | Semi-structured Interviews. conducted during three phases:(i) the antenatal period (29 to 40 weeks) (ii) the immediate postnatal period (1-2 months after birth) (iii) the late postnatal (4 months after birth) | Thematic analysis | (i) Spiritual perspective (ii) Expression of religious requirements (iii) Perceptions of healthcare professional |
| Hassan, Shaima Mohamed, et al. (2020) | To further enhance our understanding of individual experiences of healthcare, health behaviour and healthcare practices and to ultimately improve the management and provision of health services for Muslim women | December 2014- June 2015 (United Kingdom) | Snowball sampling starting with identifying key contacts in a maternity care unit | Healthcare Professionals (HCPs) from a large maternity care unit (n=12). | Semi-structured interviews | Thematic Analysis | (i)HCPs perceptions about Muslim women (ii) HCPs understanding and awareness of religious practices (iii) HCPs approaches in addressing and supporting Muslim women’s religious needs (iv) Importance of training in providing culturally and religiously appropriate woman-centred car |
| Henry, et al. (2020): 437-447.‏ | To investigate how premigration experiences, conceptions about pregnancy and childbirth, health literacy, and language skills influence access to health care, experiences of health care, and childbirth. | August -September 2017 (Germany) | Purposeful sampling via personal contacts of the interviewer, recommendations from  social workers, referrals among refugees, or in the waiting room at the refugee clinic | Arabic-speaking refugee women (n=12). Syrian (n=9), Iraqi (n=2), Palestinian (n=1) | Semi-structured interviews | Content analysis | (i) Perceptions of health-care needs (ii) Healthcare seeking (iii) Health-care consequences (iv) Health-care experiences during pregnancy and childbirth in Germany (v)Compensation mechanisms. |
| Herrel, et al. (2004) | To obtain information to develop culturally sensitive health education materials | During July 2002 (United States) | Purposeful sampling through a female Somali Community Health Worker | Somali Immigrant women (n=14) | Focus Group interviews | Not sated | (i) overall childbirth experience (ii) issues of labour and delivery (iii) childbirth education needs (iv) sources of information (v) decision making (vi) use of interpreters (vii) prenatal visits (viii) formats for health education. |
| Hill, et al. (2012) | To describe Somali immigrant women’s health care experiences and beliefs regarding pregnancy and birth | Not stated (United States) | Convenience sampling | Somali Immigrant women (n=18) | Focus Group interviews | Content analysis | (i) pregnancy as a natural experience for women, (ii) value and relevance of prenatal care, (iii) lack of control and familiarity with delivery in the United States, (iv) balancing the desire to breastfeed with practical concerns and barriers, (v) discomfort with mental health issues, and (vi) challenges in the health care system. |
| Konje, et al. (2021) | To explore and describe the views and experiences of Somali migrant women on accessing care before and during pregnancy in the UK and the factors that influence these, and on childbirth | Not stated (United Kingdom) | Convenience and purposeful sampling then snowball sampling via Somali community centres | Somali immigrant women (n=16) | a combination of focused group discussions an individual semi-structured interviews | Grounded theory | (i) positive attitude of community midwives and availability of community services (ii) language difficulties and ineffective communication (iii) lack of cultural awareness and preconceived ideas (iv) need for continuity of care and more resources (v) personal and community and religious influences, views and experiences of other Somali women. |
| Lukin, et al. (2023) | To describe Syrian women’s experiences of being pregnant and receiving care at antenatal clinics in Sweden for the first time after migration. | Not stated (Sweden) | Purposeful sampling via an antenatal clinic and Arab parent educational group | Syrian migrant women (n=11) | Individual interviews | Phenomeno-logical method | (i) It was important to feel welcomed and to be treated like an equal (ii) A good relationship with the midwife strengthened self-confidence and trust (iii) Previous experience of pregnancy and care influenced the experience the  care received |
| Missal, et al. (2016) | To explore Somali immigrant new mothers’ experience of childbirth in Minnesota | Not stated (United States) | Snowball sampling | Somali Immigrant women (n=12) | Semi-structured interviews | Ethno-nursing analysis | (i) the limitations of support due to separation from family (ii) the importance of cultural and religious practices (iii) the desired relationships with nurses (iv) the fear of Caesarean section (v) the value of education for Somali women (vi) views on postpartum blues/depression |
| Ny, et al. (2007) | To describe Middle Eastern mothers' experiences of the maternal health care services in Sweden and the involvement of their partners | February-April 2005 and 2006 (Sweden) | Purposeful sampling via antenatal clinics, Arabic-speaking local community, and schools for immigrants | Middle Eastern mothers (n=25) | a combination of focused group discussions an individual semi-structured interviews | Content analysis | (i) Access to the professional midwife (ii) Useful counselling (iii) Stable motherhood in transition (iv) Being a family in a different culture |
| Qureshi, et al. (2013) | To describe the comparative birthing experiences of immigrant Pakistani women in two different contexts: Pakistan and the United States | 2006-2009 (United states) | Purposeful sampling followed by snowball sampling via two local mosques | Pakistani immigrant women (n=26) | Individual interviews | Ethnographic analysis | (i) Significance of Collective Support in Early Pregnancy (ii) Contrasting Perception of Support from In-Laws  and Own Family (iii) Lack of Familiarity With the U.S. Health Care  System and Financing of Health Care Services (iv) Contrasting Cultural Expectations, Beliefs, and  Practices in Pakistan and the United States (v) Emergent Changes in Patterns of Decision-Making and Gendered Roles |
| Reitmanova, et al. (2008) | To document and explore the maternity health care needs and the barriers to accessing maternity health services from the perspective of immigrant Muslim women living in St. John’s, Canada | Not stated (Canada) | purposeful sampling via a local mosque and a Muslim mailing list, then snowballing via the local Muslim community | Muslim Women (n=6) | Individual interviews | Content analysis | (i) Pregnancy phase (ii) Labor and delivery phase (iii) Postpartum at home phase (iv) Barriers to maternity care |
| Simpson, et al. (2008) | To explore the experience of Muslim women through descriptions of their encounters with health care providers in a rural area | Not stated (United States) | Purposeful sampling via a local mosque | Muslim women (n=7) | Individual interviews | Phenomeno-logical analysis | (i) Perceived power of the provider (ii) Religiously defined gender relations (iii) being a stranger in the U.S. health care system. |
| Straus, et al. (2009) | To examine cultural and social aspects of childbirth, and to determine how they intersect with the needs and experiences of Somali women in the UK | Not stated (United Kingdom) | Purposeful sampling via Somal women community groups | Somali women (n=8) | Narrative interviews | Thematic analysis | (i) Circumcision (ii) Communication (iii) Cultural aspects to care (iv) Pressures arising from migration experience |
| Utne, et al. (2020) | To explore Somali women’s experiences of antenatal care in Norway | November 2017 and April 2018 (Norway) | Snowball sampling | Somali women (n=8) | Semi-structured interviews | Thematic analysis: systematic text condensation | (i) when care was provided in a way that gained their trust, they made better use of the available health services (ii) the importance of continuity of care and of sharing commonalities with the caregiver (iii) a need for accessible information, specifically tailored to the needs of Somali women (iv) how culturally insensitive caregivers had a negative impact on the quality of care |
| Wallmo, et al. (2020) | To identify and describe Somali women’s lived experience of birth giving in Sweden | Not stated (Sweden) | Purposeful and snowball sampling | Somali women (n=7) | Individual interviews | Interpretative phenomenological analysis | (i) Being recognised and confirmed as a woman (ii) Communication is important for the women’s independence (iii) Something naturally becomes unknown and complicated |
| Wojnar, et al. (2015) | To explore the perspectives of Somali couples on care and support received during the perinatal period in the United States | Not stated | Purposeful sampling and snowball sampling via community educational centre for immigrants | Somali couples: women (n=26), men (n=22) | Semi-structured interviews | Descriptive phenomenological analysis | A-Navigating through the conflicting values, beliefs, understandings, and expectations that infiltrated the experiences: (i) Feeling vulnerable, uninformed, and misunderstood (ii) Longing for unconditional respect and acceptance (iii) Surviving and thriving as the recipients of health care |
